# The application of homemade Neosinocalamus affinis AC in electrokinetic removal technology on heavy metal removal from the MSWI fly ash

**DOI:** 10.1038/srep39312

**Published:** 2016-12-21

**Authors:** Kexiang Liu, Tao Huang, Xiao Huang, Lin Yu, Faheem Muhammad, Binquan Jiao, Dongwei Li

**Affiliations:** 1 State Key Laboratory for coal mine disaster dynamics and control, Chongqing University, 400044, China; 2 City College of Science and Technology, Chongqing University, Chongqing, 400044, China

## Abstract

This present paper was focused on the manufacture of activated carbon (AC) and its application in the electrokinetic remediation (EKR) technology on removal of the heavy metals (HMs) from the municipal solid waste incineration fly ash. AC was produced from Neosinocalamus affinis (NF) by chemical activation with H_3_PO_4_ in N_2_ atmosphere, the effects of activation temperatures, soaking time and impregnation ratios on the adsorption capacity of AC on HMs were examined through equilibrium adsorption experiments. The AC produced under the condition of 450 °C of activation temperature, 10 h of soaking time and 1.5 of impregnation ration was applied in the EKR experiment. The addition of AC in the S3-region of the electrolyzer could effectively improve the removal efficiencies of HMs. The technical parameters of voltage gradient, processing time and proportion were further optimized in the coupled experiments, the maximum removal of Cu, Zn, Cd, and Pb was 84.93%, 69.61%, 79.57%, and 78.55% respectively obtained under the optimal operating conditions of 2 V/cm of voltage gradient, 8 d of processing time and 20% of proportion.

Rapid increase in urbanization, results in high consumption of energy and large volume of garbage. We need to concentrate on reduction of garbage. Waste incineration has become one of the main methods of waste disposal^1^,^2^. It involves thermal treatment which leads to garbage reduction, save space, eliminate variety of pathogens, also conversion of toxic and hazardous substances into harmless material^3^,^4^. However, the fly ash produced by burning garbage contains more dissolved salts and HMs^5^, which can contaminate the water and soil, it will cause environmental pollution, and thus harm to human health. Therefore, if we decrease the content of HMs in fly ash waste, mitigate its harm to ecological environment^6^,^7^,^8^; reduce the probability of environmental accidents, environmental safety issues can be solved.

Several techniques like physical separation/isolation, immobilization, flushing, toxicity reduction and phytoremediation exist for the remediation of heavy-metals^9^,^10^,^11^,^12^. However these techniques have number of limitations, i.e. incomplete metal removal, high usage of reagents and energy, low selectivity and generation of secondary waste products^13^,^14^. EKR is considered as a green situ or *ex situ* remediation technology, due to its simple handling, and low-power usage^15^,^16^,^17^. It is primarily used to remove and concentrate contaminants in the focusing area or in a reservoir solution. Moreover it has considerable potential for the treatment of HMs from highly clayey/microporus soils. Soil type, nature and concentration of contaminants, zeta potential and electrode spacing are the primary factors that affect the removal efficiencies of organic and inorganic contaminants from solid matrices while using EKR^18^,^19^,^20^,^21^. There are some inadequacies/limitations in EKR like (i) the presence of precipitation and composite anion in the electrolytic cell would increase the disorder of the HMs mobilization; (ii) long processing time; (iii) low removal of HMs^21^; (iv) poor handling of contaminant’s extraction may cause reading error or even second pollution^22^. The substances, which immobilize the HMs, can be used in EKR removal experiment. Addition of these substances could reduce the precipitation and composite anion^23^,^24^. Moreover it reduces the HMs exchange hindrance, if so, then the purpose of short processing time and increased concentration of HMs removal can be achieved. On the other hand, contaminants can be isolated from adsorbent and the resource utilization of fly ash can be realized by subsequent processing.

AC as a non-polar sorbent is one of the most widely used adsorbent in current experiment (wastewater treatment); it has wide deposits, easily available and relatively inexpensive^25^,^26^. It has a good adsorption capacity and stable chemical properties: resistant to acid, alkali, high temperatures, high pressure and water invasion, it could also be used repeatedly by activation and regeneration. While AC is efficient absorbent for organic compounds as compared to inorganic HMs e.g. cadmium, copper, lead, chromium^27^,^28^. The fixation of HMs can be enhanced by changing the molding conditions to modify its surface properties. In most reported cases, chemical activation is preferred to getting better porous AC^29^,^30^.

The current experiment was aim to (i) explore the effect of the molding conditions (e.g. activating temperature, activating time, and activation agent ratio) on the AC adsorptive properties of HMs, and confirm the optimal molding conditions of AC (ii) enhance the removal efficiency of MSWI fly ash and shorten the processing time by EKR coupled with AC. Based on these objectives, homemade AC under the optimal molding conditions was applied in the coupling experiment to explore the enhanced removal rate of HMs. Batch tests were performed at varying: voltage gradient, treatment time and the proportion of AC. So that proper experimental parameters for the coupled tests can be obtained. After the experiments, the removal efficiencies and leaching toxicities of the HMs in the MSWI fly ash were evaluated (i) to determine the removal of the HMs and (ii) to directly measure the remediation effect of the coupled system in the samples. X-ray diffraction (XRD), scanning electron microscopy (SEM) and Fourier transform infrared spectroscopy (FTIR) were used to further analyze and understand the mechanisms of HMs removal from MSWI fly ash.

## Methods and Experiments

### MSWI fly ash Preparation and analysis

Experimental fly ash was obtained from the waste incineration site in Chongqing municipality, China. The fly ash samples were dried in a thermostatic heater at 120 °C for 2 h and then sieved by a 100-mesh sieve. While leaching toxicities of were measured by Solid waste-Extraction procedure for leading toxicity-Acetic acid solution method (HJ/T300–2007).

### Preparation and Characterization of AC

NF from Shapingba District (Chongqing province) were washed with hot distilled water to eliminate the impurities ((dust and water soluble substances) and dried under direct sunlight until they had reached a moisture of 5 ± 0.5%, then they were cut into small into small pieces, the feedstock were dried in an oven for 18 h at 80 °C, and further sieved to obtain particles size lower than 80 meshes prior their activation. The sieved raw materials was mixed with H_3_PO_4_ (85%) at the designed impregnation ratio (g/g, H_3_PO_4_/carbon) during desired soaking time (h) with occasional stirring, later, the samples were heated at 70 °C for 6 h to removal excess moisture. As following step, the resulting AC were then placed in a furnace and chemically activated at the designed temperature for 1 h, the heating rate was 10 °C/min and the highly pure nitrogen (N_2_) flow rate was 200 ml/min during the activation process. After that, the product was cooled down to room temperate under nitrogen flow and thoroughly washed with hot deionized water until neutral pH. At the end, the obtained powder was then dried at 80 °C until constant weight and kept in hermetic bottle for subsequent use.

The surface topography and qualitative features of AC sample was observed by scanning electron microscope (SEM) (VEGA II LMU, TESCAN, The Czech) at various magnifications, the sample was gold coated prior to SEM observation, and the processing time is 10 s. Fourier transform infrared spectroscopy (FTIR) was employed to the samples before and after the test, the presence or absence of certain chemical functional groups in the samples was analyzed with the aid of Nicolet iS5 FT-IR spectrometer, the resolving power is 2 cm^−1^, scan range is 400–4000 cm^−1^, and scan number is 16 times. X-ray diffraction (XRD) spectroscopy was applied to reveal the crystal structure of the studied samples, Shimadzu XRD-6000 X-ray diffractometer was used for this purpose, the continuous scanning range is 10–80°, scanning step width is 0.02°, the tube voltage is 40 kV, tube current is 80 mA. Boehm titration is one of the most widely used methods to quantify the basic and oxygenated acidic surface groups on AC. It is assumed that: only strong acidic groups were neutralized by NaHCO_3_; both strong and intermediate acidic groups were neutralized by Na_2_CO_3_; and weak, intermediate and strong acidic groups were all neutralized by NaOH. In this experiment, 0.2 g of resulting AC samples was mixed in a closed polyethylene flask with 100 ml of a 0.01 mol/L aqueous reactant solution (NaOH, or Na_2_CO_3_, or NaHCO_3_). The mixtures were shaken for 24 h at 150 rpm, the flasks were left on bench for 8 h to let the carbon particles settle down, and then the suspensions were filtered, 10 ml of the filtrate was pipetted and back titrated with HCl (0.01 mol/L). The results were expressed as H^+^ equivalents per gram of sample.

### Equilibrium adsorption experiments

The batch method was conducted to investigate the effect of forming conditions on adsorption of metals on NFAC. Stock solution was prepared by dissolving each metal salt in deionized water, salts used were zinc chloride for Zn (II), copper chloride for Cu (II), cadmium chloride for Cd (II), lead chloride for Pb (II), the amounts of these four metal salts were 200 mg/L, 30 mg/L, 15 mg/L, 50 mg/L, the additions of HMs were determined by the results of the leading toxicity of raw MSWI fly ash. Considering the high content of Ca(II), Al(III), Na (I) in the raw samples and the competitive adsorption between these HMs, 300 mg/L of CaCl_2_, 150 mg/L of AlCl_3_ and 100 mg/L of NaCl were also added, the pH was adjusted to 5 ± 0.1 using 0.1 M solutions of HCl and NaOH. 100 ml solution was placed in a 200 ml conical flask with 0.1 g AC and was agitated at a speed of 120 rpm in a thermostatic shaker bath at 30 °C for 24 h. The initial concentration of metal ions (Zn^2+^, Cu^2+^, Cd^2+^, and Pb^2+^) and corresponding concentrations after fixed time periods were measured by atomic absorption spectrophotometry (SK-20002B). The adsorption capacity (mg/g) Q of the AC from the simulated solution in the equilibrium adsorption experiments was calculated using the following Eq. (1):


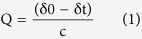


where δ_0_ and δ_t_ denote the initial and equilibrium concentrations of the adsorbates, individually, c was the quality (mg) of AC in per liter solution.

### EKR removal experiment

**Figure 1:**
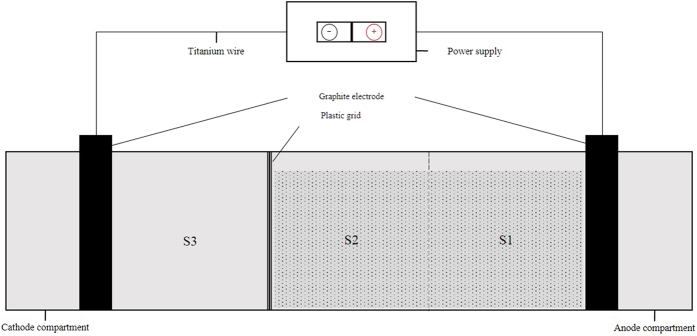
The schematic of the electrokinetic test device.

The EKR experiments were conducted in rectangular toughened glass instrument, the test setup consisted of an EKR cell, two electrode compartments, plastic grid coated by 200-mech nylon mesh, a power supply and so on. Figure 1 showed the schematic of the EKR testing device used for this study.

The chamber (150 mm*100 mm*100 mm) was evenly divided into three regions, for the coupled experiment, S2 and S3 is separated using plastic grid, as for the simple EKR, the chamber holding MSWI fly ash samples was an integral whole. The graphite electrodes (10 mm*100 mm*100 mm) acted as anodes and cathodes were placed at the end of the sample chamber. Only deionized water was poured into the cathode compartment and anode compartment during the test, the operating parameters of the three types of batch experiment are shown in Table 1. To further elevate the removal efficiencies of HMs in the fly ash samples and obtain the optimum performance of the coupled experiment, three factors including the voltage gradient, the processing time and the proportion of activated charcoal, each at three different levels, were considered to design, the exclusion rates of the corresponding HMs under the designed experimental conditions (L_9_ (3^4^)) were shown in Table 2.

### Leaching toxicity analysis

The leaching toxicities of Zn, Pb, Cu and Cd were determined based on the toxicity characteristic leaching procedure (HJ/T300–2007). The MSWI fly ash sample (10 g) mixed with 200 mL of 1.725% concentration acetic acid was shaken on a rotary shaker at approximately 30 r/min for 18 h. Then, the filtrate was adjusted by nitric acid and was measured by ICP-OES to determine the concentration of the HM elements. The removal rate of the HMs in a fly ash sample was calculated as Eq. (2):


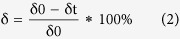


where δ is the HMs removal rate (%) in the fly ash sample, δt is the concentration of HMs (mg/L) in the sample after the processing time t, and δ0 is the initial HMs concentration (mg/L) in the original sample.

## Results and Discussion

### Effects of the molding conditions on the adsorption capacity of AC

#### Effects of the activation temperature

**Figure 2:**
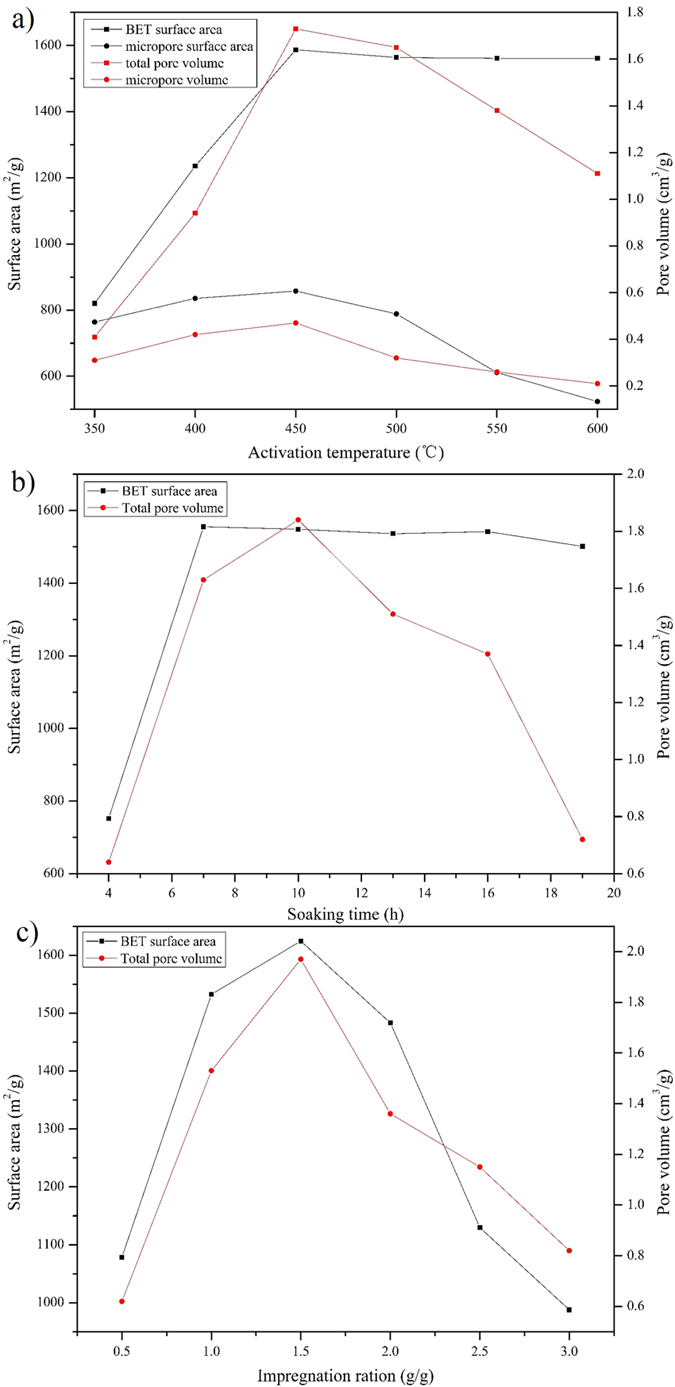
Physical structure properties of AC under different molding conditions (**a**) activation temperature, (**b**) soaking time, (**c**) impregnation ratio.

**Figure 3:**
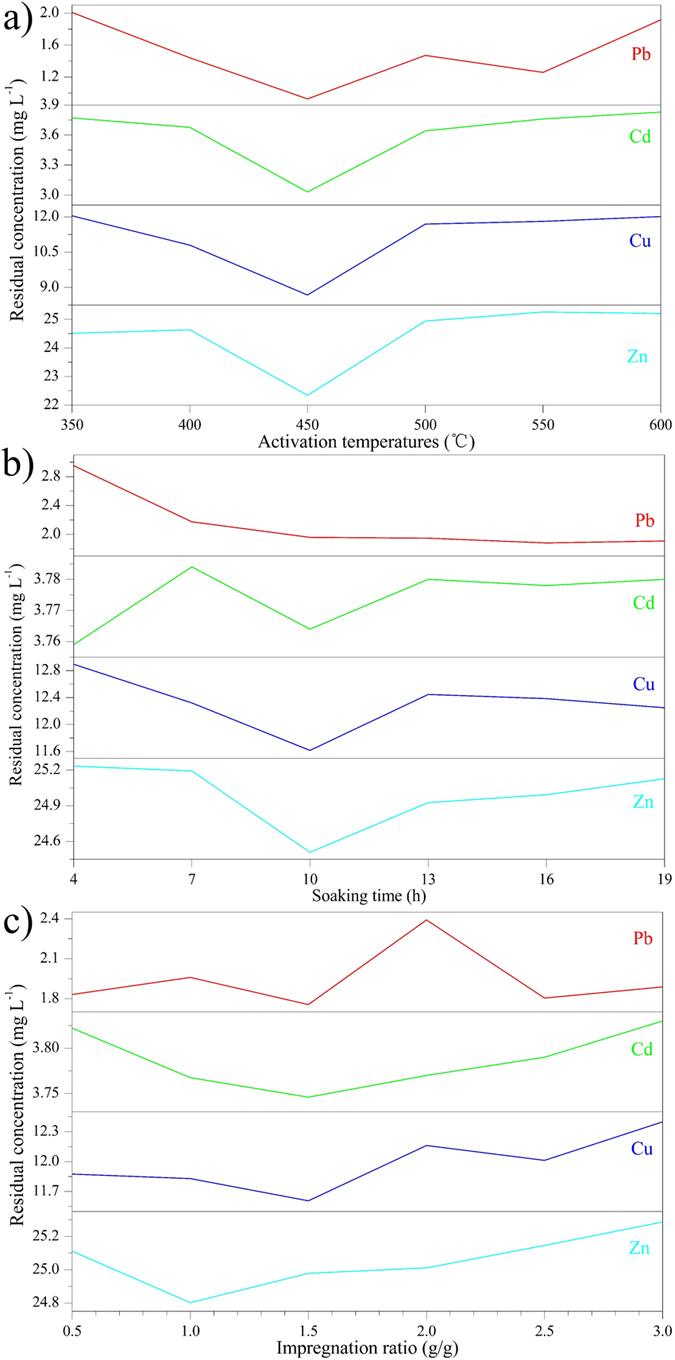
Adsorption capacity of AC under different molding conditions (**a**) activation temperature, (**b**) soaking time, (**c**) impregnation ratio.

In the equilibrium adsorption experiments, heavy metal salts were used as the absorbates, the detail results were shown in Table 3. The batch adsorption behaviors of resulting AC with varied activation temperature were first investigated, the temperature range of carbonization employed in the present study was 350–600 °C, the impregnation ratio was set to 1.0 and the soaking time was 10 h. From the Fig. 2a physical structure properties of AC at different activation temperatures, it was clear that with the rising of temperature, the BET surface area increased swiftly at the beginning, then slowly declined, and finally reached the stable status. While the micropore surface area, total pore volume and micropore volume presented the trend of first increase then decrease. BET surface area and total pore volume was growing rapidly at 350–450 °C, and reached the maximum value 1587.32 m^2^/g and 1.73 cm^3^/g at 450 degrees, respectively. While the total pore volume and micropore volume gradually diminished when the temperature exceeds 500 °C. It can be seen from the Fig. 3a that all the residual concentration reached the minimum value at 450 °C, when the final heat treatment temperature exceeded 450 °C, the residual concentration tended to enlarge with the increase of carbonization temperature. While from 350 °C to 450 °C, the adsorption capacity of four HMs was all increased apparently. This may due to the fact that the pores of AC are mainly caused by the volatilization of organic matter, when the activation temperature was below 450 °C, the degree of activation was not complete, the specific surface area was small, the micropore and mesoporous was less. So the adsorption capacity of HMs was weak, and the residual concentration was high. In the early stage, the BET surface area and total pore volume increased with the increase of activation temperature, the adsorption capacity gradually enhanced, and the residual concentration tended to drop off over temperature. When the activation temperature exceeded 450 °C, increasing the temperature will not significantly change the specific surface area, but the high temperature will wreck micropore which have great influence on the adsorption capacity of AC, the levels of residual HMs would have a trend of gradual increase when the temperature exceed 450 °C^31^,^32^.

#### Effects of the soaking time

To explore the effects of the impregnating conditions raw materials were added to H_3_PO_4_ solution at 1.0 impregnation ratio, and maintaining the mixing for different periods of time (4 to 19 h), the treated samples were then carbonized at 450 °C. From the Fig. 2b physical structure properties of AC with different soaking time, the BET surface area slightly decreased after the first rapid increase, the total pore volume rapid increased firstly but decreased subsequently. At 7 h, the BET surface area achieved the maximum value, 1555.54 m^2^/g. The maximum value 1.84 cm^3^/g of total pore volume was observed at 10 h. From Fig. 3b, an obvious decrease was found in the residual concentration of Cu, Zn and Pb from 4 h to 10 h, the former two element attained the minimum value at 10 h, and the last element showed a minor change in subsequent experiments (10 to 19 h), while Cu Zn tended to increase. For Cd, Soaking time showed little effect, the residual concentration was just around 3.77 mg/L. This maybe that in the initial phase, with the extension of the soaking time, phosphoric acid and raw materials were combined gradually, the decomposion and escape of organic matter in the bamboo formed a certain pore, which increased the total specific surface area and promoted the adsorption capacity of resulting AC, so in this phase, there is a gradually decreasing trend of the residual HMs. But with the continued increasing of immersion time, the binding ability between the activator and raw materials was stronger, which prevent the decomposition of organic matter in the raw materials^32^,^33^, the decrease of the total specific surface and total pore volume weakened the adsorption capacity of the resulting AC.

#### Effects of the impregnation ratio

The impregnation ratio of phosphoric acid: dry precursor has also been reported to be important in determining the pore structure of resulting carbons. The effects of the ratio on the adsorption capacity of the resulting carbons were also explored, the activation temperature was 450 °C, the soaking time was 10 h and the impregnation ratio was from 0.5 to 3. From the Fig. 2c physical structure properties of AC with different impregnation ratios, the BET surface area and the total pore volume had the same change tendency, briefly increases initially and decreased rapidly subsequently. At the impregnation ratio of 1.5, both reached the maximum value, 1548.38 m^2^/g and 1.92 cm^3^/g, respectively. As can be seen from Fig. 3c, the residual concentration of Cd and Cu exhibited a decrease when the ratio from 0.5 to 1.5, and then an increase when the ratio exceed 1.5. Zn show a similar trend, while the minimal value appeared earlier compared with the former two element. Pb was slightly complicated, it seemed the adsorption capacity of Pb was sensitive to impregnation ratio, but the minimal value also appeared at the ratio of 1.5. This may due to the fact that, when the impregnation ratio was relatively low, the impregnation of raw materials by phosphoric acid is not sufficient, the specific surface area and pore volume was small, the adsorption capacity of HMs was week, the specific surface area and the adsorption capacity of HMs increased with the increase of impregnation ratio in the first phase^32^,^34^,^35^. But superfluous activator could prevent the shrinkage of raw materials, which will cause the reduction of the special area and the diminution of the adsorption capacity.

The carbon used in the following experiment was manufactured in the condition of 450 °C of activation temperature, 10 h of soaking time and 1.5 of the impregnation ration. BET surface areas was 1637.42 m^2^/g, pore volume was 2.14 cm^3^/g, and the surface groups on resulting carbon were shown in Table 4. The equilibrium adsorption capacity for Cu, Zn, Cd and Pb was 117.96 mg/g, 73.72 mg/g, 21.01 mg/g, 35.97 mg/g, respectively.

#### The contrast experiments

**Figure 4:**
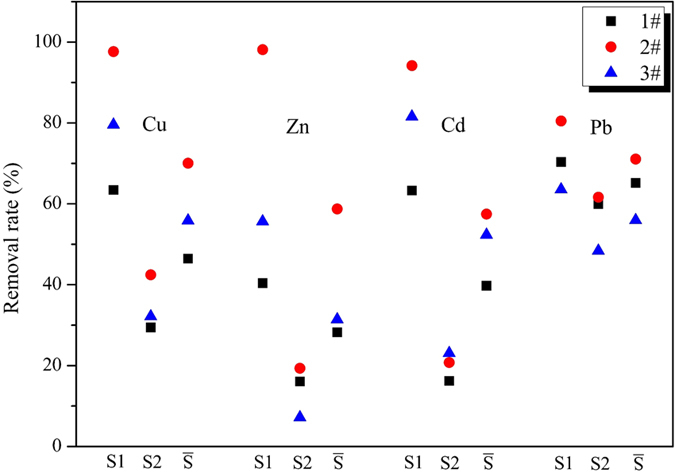
Removals rate (δ) of HMs in the S1 and S2 regions.

Three types of batch tests (Table 1) were conducted to measure the effect of the coupled system on the removal of HMs, the analysis of leaching toxicity removal efficiency was conducted at the end of the experiment; four sampling points distributed evenly in S1, S2 region, and eventual outcome was averaged as the index of the removal efficiency of HMs. As shown in Fig. 4, significant difference on the removal rate (δ) among three systems was found, the δ of HMs in 2# was significantly elevated in the experimental region compared with the other two groups. The δ values of four HMs in the coupled process in the S1 region were always much higher than 1# and 3# group, and the 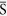 (average δ) values were similar to this. As δ value shown in Fig. 4, the difference of δ in S1, S2 region was significant, δ values of S1 in 2# were all above 80%, whileδ of S2 in all three types was relatively small. The result was evident, for each HMs, δ value of S2 is all much smaller than that in S1.

The underlying reasons for this phenomenon might be that, oxygen-containing functional groups on the surface of AC could adsorb Cd^2+^, Pb^2+^, Cu^2+^, Zn^2+^ and their complex anions, which could reduce migration resistance of metals and disorders of the metal ion migration, consequently, the δ value in the complete experimental region was improved. Due to the small voltage gradient or short processing time, EKR remediation in S2 weren’t inadequate, so δ value in this region was much smaller than S1.

**Figure 5:**
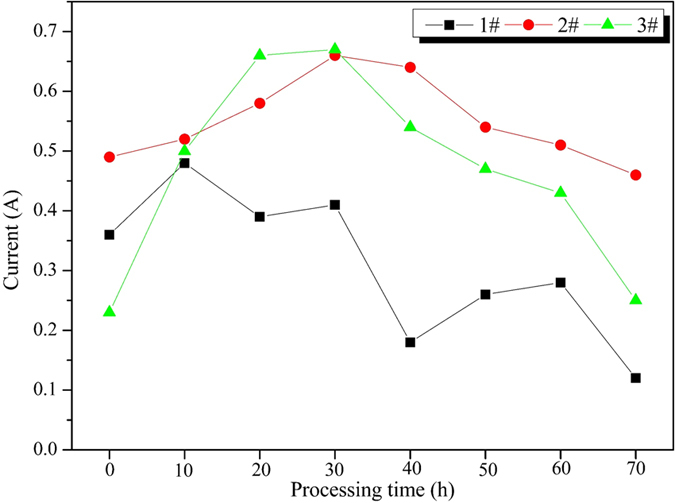
Current changing of the contrast experiments.

The trend of the current in the batch coupled tests was shown in Fig. 5. The general trends of the current under three conditions showed a similar pattern: firstly increase and then decrease. The main reason of the initial current increased was the elevated concentration of the dissolved free ions and the continued release of the metal irons, the decrease of the terminal current was primarily due to the concentration polarization, the increase of the resistance caused by the production of some precipitates^24^. Current in 3# was a typical curve of increase first and then decrease, current in 2# was a similar one, while the value is relatively concentrated, and the change range is not severe. If there is only physical adsorption, the pore structure of AC will be blocked by the precipitates, most of precipitates were nonconductive, then the conductive capacity of AC will be greatly reduced, the current will show a significant change. The relative stable current may indicated that chemical adsorption existed in the adsorption of AC on HMs, which can reduce the production of the precipitates and decrease the resistance of the electron mobility. Current in 1# was a little complex, it seem like a combination of two curves of firstly increase and then decrease, this demonstrated the unstable current, the probable reason was the massive complex anions increase the disorders of the metal ion migration.

#### Optimum analysis in the coupling experiment

**Figure 6:**
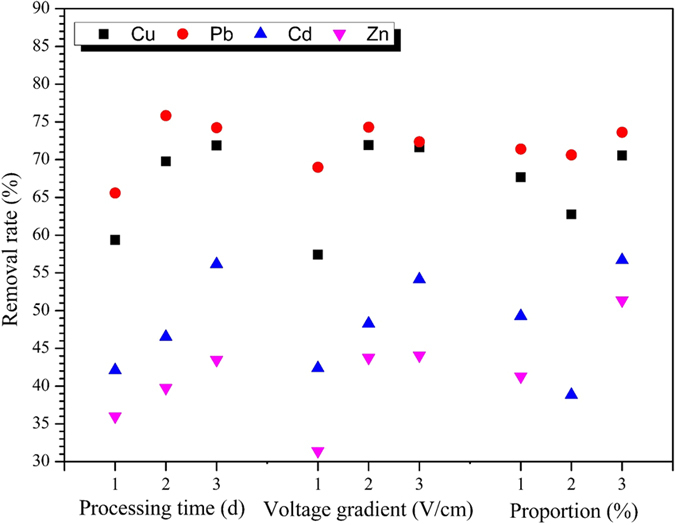
Mean removal rates of HMs in the orthogonal experiment.

In order to determine the optimum parameters levels for EKR coupling, an orthogonal experiment was performed. The parameters included in this orthogonal experiment were processing time (factor A), voltage gradient (factor B) and proportion of activated charcoal (hereafter referred to as proportion (factor C)), each factor replicated three times having three levels. The exclusion rates of the corresponding HMs under the designed experimental conditions (L_9_ (3^4^)) shown in Table 2. The maximum removal of Cu (82.48%), Cd (62.88%) and Pb (77.82%), was noted at processing time of 8d, voltage gradient of 1.5 V/cm and 10% of proportion. While corresponding maximum values of Zn (58.75%) were observed, when 3d of processing time, 1.5 V/cm of voltage gradient and 20% of proportion were used. The mean δ under the EKR coupling experiment with three experimental factors at three different levels was shown in Fig. 6. The range analysis was adopted to compare the significances of each experimental factor at each level and confirm the optimum technological conditions of the EKR coupling, and the results were shown in Table 5.

For the removal rate of Cu element, the maximum value (71.87%) was obtained at level 3 in factor A, 71.94% was achieved at the level 2 in factor B, 70.56% was observed at level 3 in factor C. Thus, the optimum combination for the removal of copper was A3B2C3. In the method of range analysis, the greater (R) value, the higher degree of the influence. The R of factor A, B and C was 12.51, 14.53 and 7.81, the R of voltage gradient was the biggest one, which indicated voltage gradient was the key factor that affected the copper removal efficiency. Following the same way, the key factor and the optimum combination of Zn, Cd and Pb can also be obtained, For Zn, Cd and Pb, the critical factor was C, C and A, respectively. The optimum combination was A3B3C3, A3B3C3, and A2B2C3, individually.

The optimum processing time for Cu, Cd and Zn removal rate was 8d, for Pb the removal rate at 5 d and 8 d was close (5 d: 75.83%, 8 d: 74.24%), therefore, the optimal voltage gradient was adjusted to 8 d. The removal rate of Cu, Pb at 1.5 V/cm and 2.0 V/cm was found to be approximate, considering the optimum voltage gradient for Zn, Cd was 2 V/cm, the optimum voltage gradient was set to 2 V/cm. It was easy found that proportion of 20% was the optimal dosing ratio for all HMs. It is concluded that the optimum voltage gradient, processing time and proportion was 2 V/cm, 8 d and 20%, respectively. And under this condition, the removal rate of Cu, Zn, Cd, and Pb was 84.93%, 69.61%, 79.57%, and 78.55%, individually. It was clear the removal rate was significantly improved.

#### XRD analysis

**Figure 7:**
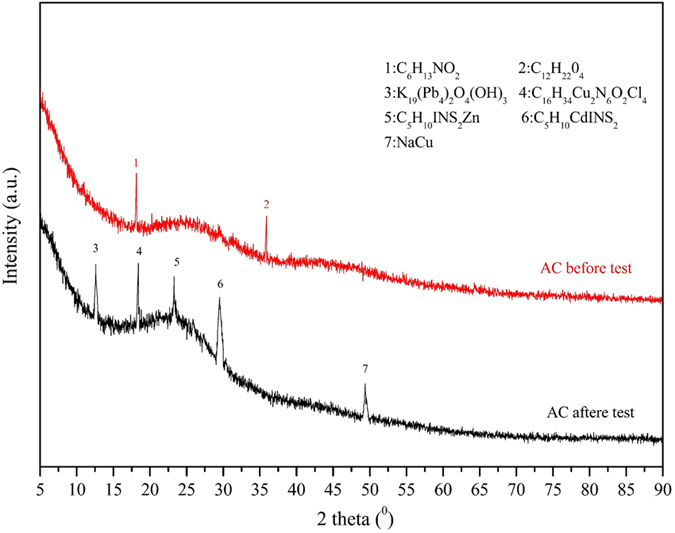
X-ray diffraction pattern of the AC before and after the experiment.

X-ray diffraction analysis is an effective method for the qualitative analysis of crystal structure. In order to observe the changes of composition of AC before and after the test, XRD spectra were recorded. As can be seen from the Fig. 7, besides, the same broad peak at about 25° which corresponded to the (002) of graphite, there are also different apparent peaks in the two XRD pattern. As shown in Fig. 7, C_6_H_13_NO_2_ and C_12_H_22_O_4_ was detected in the original AC, this may be the original compositions of Neosinocalamus affinis, or the reaction product of raw material and activator.

After coupling experiment, more complex inclusion like C_5_H_10_INS_2_Zn, C_16_H_34_Cu_2_N_6_O_2_Cl_4_, C_5_H_10_CdINS_2_, K_19_(Pb_4_)_2_O_4_(OH)_3_, NaCu present in the structure of the AC. The existence of those complicated organometallic compound may be due to the reaction between metal irons and AC, surface functional groups of AC interacted with metal irons and formed new phase. In the EKR remediation environment metal ions shifted to metal salt, and those inorganic substance was probably adsorbed physically by AC.

#### SEM analysis

**Figure 8:**
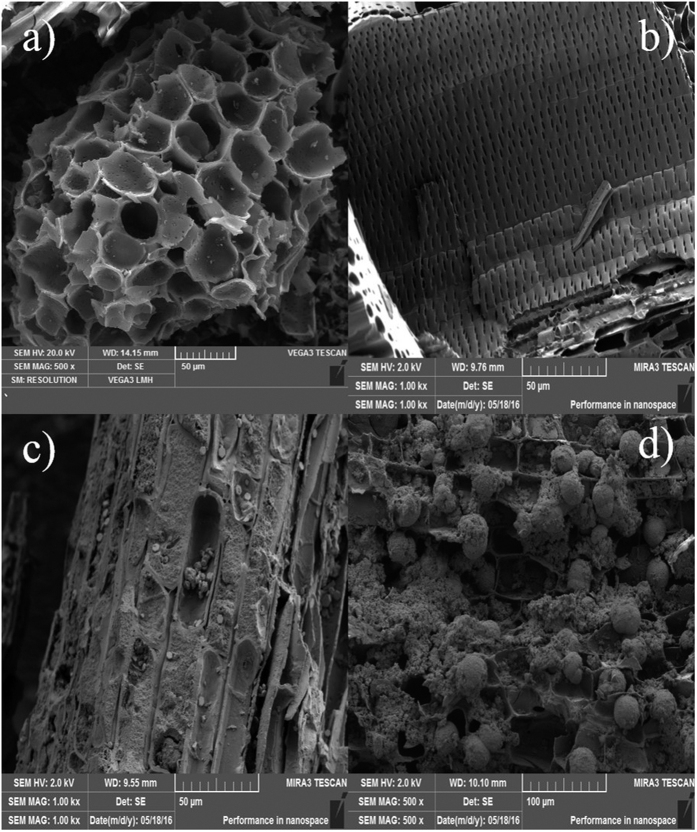
SEM images of AC (**a**) (**b**) from raw AC, (**c**) (**d**) from used AC.

It can be clearly seen from the scanning electron micrographs Fig. 8(a,b), the pore structure of AC was developed, a large number of pores distributed on the surface of the resulting AC. The well-developed pore structure similar to honeycomb were distributed on the original AC surface which was smooth, pore edge was clearly visible, the hole wall was smooth, on which there were varying amounts of pores distributed, as well as part of the site of fracture or collapse, forming larger pore size. NF were corroded in the acidic environment provided by the phosphoric acid activator, the pore wall became thin after high temperature activation^29^.

After the coupling experiment, Fig. 8(c,d) showed that the AC surface was covered with a large amount of flocculation, the void structure was hardly observed, large pore and micro pore and honeycomb porosity were all blocked, hole wall became blurred and rough, the corrosion of the structure was serious, even denudation phenomenon appeared in partial structure. In the process of EKR remediation, the alkaline environment generated by electrolysis in cathode may cause a corrosive effect on the structure of resulting AC. The HMs in fly ash migrated to the cathode under the electrostatic force, reacted with the OH^−^ and generated precipitation which was absorbed by AC.

#### FTIR analysis

FTIR analysis is usually considered to be a qualitatively or semi-quantitatively method to determine the chemical elements present and analyze the structural characteristics and functional groups of organic, in organic and compound molecules. Through the comparison and the characteristic absorption peaks in the spectrogram, the change of the functional groups on the surface of adsorbent can be clearly determined.

**Figure 9:**
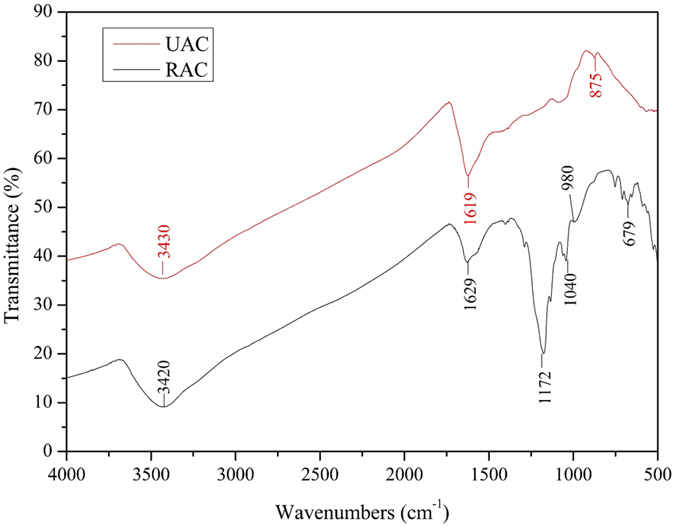
FTIR spectra of AC before and after the tests.

FT-IR spectra of the virgin and treated AC were depicted in Fig. 9. It could be noticed that the peaks at ~3430, ~1580 cm^−1^ appeared for all the AC samples, which suggested that they possessed similar groups on their surface. The AC-H_3_PO_4_ characteristics bands at 1040 cm^−1^ assigned to aliphatic phosphate stretch ν (-P-O-C-), 980 cm^−1^ (s) was assigned to ν (-P-O-P-) stretching. Broad band at 1000–1300 cm^−1^ was usually found with oxidized carbons and had been assigned to C-O stretching in acids, alcohols, phenols, ethers and esters, it was also a characteristic of phosphorus and phosphor-carbonaceous compounds present in the phosphoric acid AC. The shoulder at 1080–1065 cm^−1^ was ascribed to P^+^-O^−^ in acid phosphate eaters and to the symmetrical vibration in polyphosphate chain P-O-P. 3430 cm^−1^ (broad) assigned to boned and non-boned hydroxyl groups ν (-OH), 1570 cm^−1^ (s) assigned to the C=C stretching vibration. The peak at 1172 cm^−1^ was usually assigned to C–O stretches in lactonic, ether and phenol groups, 679 cm^−1^ was usually assigned to C-O-C bending, C=N stretching at 875 cm^−1^.The FTIR spectroscopy result indicated that the produced carbons are rich in surface functional group.

Figure 9 gave a clear distinction of difference in surface functional groups of the two AC. After the EKR experiment, -OH in the virgin AC at 3420 cm^−1^ was shifted to 3430 cm^−1^, and C=C in the virgin AC at 1580 cm^−1^ was shifted to 1570 cm^−1^. C-O at 1172 cm^−1^, C-O-C at 679 cm^−1^ and –P-O-C- at 1040 cm^−1^ was disappeared, The reason for the disappearance of peaks should be the functional groups grafted on AC have been replaced in the complex electrokinetic environment, on the other hand, as can be seen from the SEM images, after test AC was covered with a large amount of flocculation, those flocculation block the detection of the original functional groups. The appearance of C=N at 875 cm^−1^, which may reflected the adsorption of some inorganic pollutant. Those changes directly reflected chemical adsorption of AC on heavy metals was involved in EKR remediation experiment, and functional groups on the surface of carbons were the key.

## Conclusion

In this present study, AC was produced from NF by chemical activation with H_3_PO_4_ in N_2_ atmosphere. With the increase of activation temperature, soaking time and impregnation ratio, the adsorption capacity of AC on HMs was gradually increased at first and then decrease. The optimal conditions are adjusted to be activation temperature of 500 °C, soaking time of 10 h, impregnation ratio of 1.5, giving the equilibrium adsorption capacity for Cu, Zn, Cd and Pb was 117.96 mg/g, 73.72 mg/g, 21.01 mg/g, 35.97 mg/g, respectively. The addition of AC in the S3-region of the electrolyzer could effectively improve the remediation efficiencies of organic pollutants. The maximum removals of Cu, Zn, Cd and Pb were noted under different experimental conditions. It was found that the changes of the factors affected the removal of HMs to varying degrees. The voltage gradient had significant effect on the removal of Cu, while the proportion on the Zn and Cd, and the processing time on Pb produced a more positive impact on the final efficacy. The optimal test conditions were estimated to be 2 V/cm of voltage gradient, 8 d of processing time and 20% of proportion with obtaining the corresponding removal of Cu of 84.93%, Zn of 69.61%, Cd of 79.57% and Pb of 78.55% respectively. The carbon surface of the original resulting AC showed honeycomb-like morphology, the large surface area and multi-walled AC guaranteed the high adsorption capacity and numerous reaction sites. The change of the chemical composition of AC before and after the experiment demonstrated the physical and chemical adsorption of HMs existed in the whole process of EKR remediation, causing the high removal rates of HMs. The result of FTIR spectroscopy indicated that the homemade AC was rich in the surface functional groups playing an important role in the adsorption of HMs.

## Additional Information

**How to cite this article**: Liu, K. *et al*. The application of homemade Neosinocalamus affinis AC in electrokinetic removal technology on heavy metal removal from the MSWI fly ash. *Sci. Rep.* **6**, 39312; doi: 10.1038/srep39312 (2016).

**Publisher's note:** Springer Nature remains neutral with regard to jurisdictional claims in published maps and institutional affiliations.

## Figures and Tables

**Table 1:** The experimental conditions of three types of batch tests.

Type	Sample Chamber			Electrode electrolyte		Experimental conditions	
	S1	S2	S3	Anode	Cathode	Voltage gradient	Processing time
1#	Fly ash	Fly ash	Deionized water	Deionized water	Deionized water	1 V/cm	3d
2#	Fly ash	Fly ash	Activated carbon	Deionized water	Deionized water	1 V/cm	3d
3#	Fly ash	Fly ash	Fly ash	Deionized water	Deionized water	1 V/cm	3d

**Table 2:** The design of orthogonal tests of the coupled system (three factors with three levels, L_9_(3^4^)).

Test No	Processing time	Voltage gradient	Proportion	Removal rate (%)			
				Cu	Zn	Cd	Pb
1	1(3d)	2	2(15%)	63.30	19.20	24.53	74.00
2	2(5d)	2(1.5 V/cm)	1(10%)	77.28	52.74	60.35	77.15
3	3(8d)	2	3(20%)	68.74	47.35	54.75	76.35
4	2	3	1	82.48	53.33	62.88	77.82
5	3	3	2	60.22	29.07	47.77	71.41
6	1	3(2 V/cm)	3	72.91	47.99	57.86	73.48
7	3	1(1 V/cm)	1	43.30	17.75	24.65	59.23
8	1	1	3	70.05	58.75	57.48	71.08
9	2	1	2	64.74	31.46	44.23	66.48

**Table 3:** The residual concentration (mg/L) under various molding conditions.

Element		Pb	Cd	Cu	Zn
Molding conditions					
Activation Temperature ( °C)	350	2.008	3.771	12.045	24.509
	400	1.441	3.677	10.799	24.635
	450	0.927	3.031	8.677	22.339
	500	1.4715	3.642	11.691	24.941
	550	1.2585	3.761	11.806	25.256
	600	1.916	3.83	12.009	25.207
Soaking Time (h)	0.5	1.831	3.822	11.8745	25.111
	1	1.959	3.7675	11.83	24.802
	1.5	1.755	3.746	11.607	24.978
	2	2.391	3.77	12.1615	25.011
	2.5	1.804	3.79	12.012	25.146
	3	1.888	3.83	12.398	25.287
Impregnation ration (g/g)	4	2.951	3.759	12.895	25.233
	7	2.174	3.784	12.321	25.193
	10	1.959	3.764	11.615	24.509
	13	1.948	3.78	12.446	24.926
	16	1.881	3.778	12.384	24.993
	19	1.908	3.78	12.248	25.127

**Table 4:** Surface chemical characteristics of the resulting AC.

Sample	Total acidity (mmol/g)	Strong acid group (mmol/g)	Intermediate acid group (mmol/g)	Weak acid group (mmol/g)
AC	2.78	2.28	0.22	0.28

**Table 5:** The range analysis of Cu, Zn, Cd, Pb element.

	Cu			Zn			Cd			Pb		
	A	B	C	A	B	C	A	B	C	A	B	C
k_j1_	59.36	57.42	67.69	35.99	31.39	41.27	42.12	42.39	49.29	65.59	69.00	71.40
k_j2_	69.77	71.94	62.75	39.76	43.76	26.58	46.54	48.30	38.84	75.83	74.30	70.63
k_j3_	71.87	71.64	70.56	43.46	44.07	51.36	56.17	54.15	56.70	74.24	72.37	73.64
R	12.51	14.53	7.81	7.48	12.67	24.79	14.05	11.76	17.85	10.24	5.30	3.00
Optimal level	A3	B2	C3	A3	B3	C3	A3	B3	C3	A2	B2	C3
order	B>A>C			C>B>A			C>A>B			A>B>C		
